# Effects of vitamin D supplementation on the outcomes of patients with pulmonary tuberculosis: a systematic review and meta-analysis

**DOI:** 10.1186/s12890-018-0677-6

**Published:** 2018-06-28

**Authors:** Hong-xia Wu, Xiao-feng Xiong, Min Zhu, Jia Wei, Kai-quan Zhuo, De-yun Cheng

**Affiliations:** 1 Department of Respiratory and Critical Care Medicine, West China Hospital, Sichuan University, NO.37 Guoxue Alley, Chengdu, 610041 Sichuan China; 2 Department of Respiratory Medicine, Chengdu Second People’s Hospital, Chengdu, China; 3 Department of Neurosurgery, Suining Municipal Hospital of TCM, Suining, China

**Keywords:** Vitamin D, Tuberculosis, Meta-analysis, Therapy

## Abstract

**Background:**

Vitamin D is involved in the host immune response toward *Mycobacterium tuberculosis*. However, the efficacy of vitamin D supplementation on sputum conversion, clinical response to treatment, adverse events, and mortality in patients with pulmonary tuberculosis (PTB) remains controversial. We aimed to clarify the efficacy and safety of vitamin D supplementation in PTB treatment.

**Methods:**

We searched Medline, Embase, Cochrane Central Register of Controlled Trials, Web of Science for double-blind, randomized controlled trials of vitamin D supplementation in patients with PTB that reported sputum conversion, clinical response to treatment, adverse events, or mortality, published from database inception to November 26, 2017. This study was registered with PROSPERO, number CRD42018081236.

**Results:**

A total of 1787 patients with active PTB receiving vitamin D supplementation along with standard anti-tuberculosis regimen were included in the eight trials with different doses of vitamin D ranging from 1000 IU/day to 600,000 IU/month at different intervals. Primary analysis revealed that vitamin D supplementation increased the proportion of sputum smear and culture conversions (OR 1.21, 95%CI 1.05~ 1.39, z = 2.69, *P* = 0.007; OR 1.22, 95%CI 1.04~ 1.43, z = 2.41, *P* = 0.02), but did not improve the time to sputum smear and culture conversions (HR 1.07, 95%CI 0.83~ 1.37, z = 0.50, *P* = 0.62; HR 0.97, 95%CI 0.76~ 1.23, z = 0.29, *P* = 0.77). In the secondary analysis, vitamin D improved serum 25(OH)D, plasma calcium concentration, lymphocyte count, and chest radiograph (MD 103.36, 95%CI 84.20~ 122.53, z = 10.57, *P* < 0.00001; SMD 0.26, 95%CI 0.15~ 0.37, z = 4.61, *P* < 0.00001; MD 0.09, 95%CI 0.03~ 0.14, z = 2.94, *P* = 0.003); MD -0.33, 95% CI -0.57~ − 0.08 z = 2.57, *P* = 0.01), but had no impact on adverse events, mortality and other indicators(TB score, BMI, mean mid-upper arm circumference, weight gain, CRP, ESR, and other blood cells) (*P* > 0.05).

**Conclusions:**

Vitamin D supplementation can be considered as a combination therapy in patients with PTB.

**Electronic supplementary material:**

The online version of this article (10.1186/s12890-018-0677-6) contains supplementary material, which is available to authorized users.

## Background

Tuberculosis (TB) is a major health problem; the World Health Organization estimates that there were 10.4 million incident cases and 1.7 million deaths due to TB worldwide in 2016. Although TB is a preventable and curable disease, the high prevalence of multidrug-resistant and extensively drug-resistant TB with the pandemics of human immunodeficiency virus infection and diabetes generates further problems [1]. The duration of anti-tuberculosis treatment is long and requires multiple drugs that often have mild to severe side effects. Thus, there is an urgent need for developing novel drugs that can shorten treatment duration and combat infection with both susceptible and resistant TB strains.

Two epidemiological studies demonstrated that seasonal variations in serum vitamin D concentration were strongly related to the incidence of TB [2, 3]. A meta-analysis found that low serum vitamin D status was associated with increased risk of TB [4]. These results suggested that vitamin D supplementation is likely to have a primary preventive effect on the incidence risk as well as a beneficial effect on the anti-tuberculosis treatment.

The use of vitamin D for TB treatment started in 1849, with the observation that oil from fish liver improved appetite and strength [5]. The major circulating metabolite of vitamin D, 1,25-hydroxyvitamin D (1,25[OH]D), supports innate antimicrobial immune responses, suggesting a potential mechanism by which adjunctive vitamin D might enhance response to anti-tuberculosis therapy [6]. In recent years, vitamin D was used to treat PTB in the pre-antibiotic era. Thus far, two meta-analyses incorporating the data from trials of vitamin D supplementation as treatment in patients with PTB have been performed. A meta-analysis that included four trials did not show any improvement in the clinical parameters (mortality, sputum smear positivity, and sputum culture positivity) of vitamin D administration compared with placebo (*P* = 0.05) [7]. Another meta-analysis that included five studies (one study aimed at children) showed that vitamin D supplementation does not have any beneficial effects on improving sputum smear and culture conversion, adverse effects, and body weight [8]. Both meta-analyses had limited number of studies, sample sizes, and parameters analyzed, influencing the results. Currently, several new RCTs have been published. We conducted a meta-analysis of all published RCTs to update and further clarify the efficacy and safety of vitamin D as adjunctive therapy in patients with PTB.

## Methods

Each enrolled trial was approved by the corresponding Institutional Ethical Committee. Ethics approval and consent to participate are not relevant for systematic reviews and meta-analysis. This study was registered with PROSPERO. Findings are reported according to the PRISMA guidelines.

### Search strategies

In this systematic review and meta-analysis, we searched Medline, Embase, Cochrane Central Register of Controlled Trials, Web of Science for trials in process using the keywords “tuberculosis or tuberculoses” and “vitamin D or cholecalciferol,” with limitations in the publication type of RCTs but not in the publication language or period. We regularly updated our searches from database inception up to and including Nov 26, 2017. We reviewed the references listed in each identified article and manually searched the related articles to identify all eligible studies and minimize potential publication bias.

### Inclusion and exclusion criteria

Clinical trials were considered eligible based on the following criteria: 1) RCTs, 2) trials conducted in patients newly diagnosed with PTB who were on initial anti-tuberculosis treatment, 3) those conducted in patients aged > 16 years, and 4) those conducted in patients receiving vitamin D3 or vitamin D2 as an interventional treatment, with reported data on efficiency or safety of vitamin D supplementation. Patients had taken oral corticosteroid or other immunosuppressant therapy, or drugs known to interfere with vitamin D levels, or antituberculous therapy were excluded. Trials conducted in pregnant women; retrospective, observational, cohort, and case control studies; and congress articles were excluded.

### Outcome measures

Outcome measures consisted of efficacy assessment and safety evaluation, which were divided into primary and the secondary results. Primary outcomes included proportion of sputum smear or culture conversion and time to sputum smear or culture conversion. Secondary outcomes included serum 25-hydroxy vitamin D(25(OH)D) concentration, serum calcium concentration, TB score, chest imaging, body mass index (BMI), weight gain, mean mid-upper arm circumference, C-reactive protein (CRP), erythrocyte sedimentation rate (ESR), blood indexes (total white cell, neutrophil, monocyte, lymphocyte, and hemoglobin), adverse effects, and mortality.

### Study selection

The study selection was performed by two investigators in two phases to determine which studies are suitable. Duplicated and non-randomized controlled studies were discarded by screening titles and abstracts firstly. Secondly, accordance with the previously designed study inclusion criteria, eligible studies were extracted by reviewing full texts.

### Data extraction

Two data collectors extracted and recorded authors, publication year, registration series, study design, participants and population, demographic characteristics, baseline characteristics, details of intervention treatment (dose, frequency, routine, and duration), follow-up period, outcome measures and study results of each enrolled study in a standard form as recommended by Cochrane [9], independently. For any missing information, corresponding authors were contacted by email.

### Quality assessment

Five GRADE considerations [10] was used to assess the quality of evidence contributing to the analyses of efficacy assessment and safety evaluation. For the assessment of risk of bias in estimating the study outcomes, the Cochrane risk of bias tool [9] was used.

A third investigator was consulted to solve any disagreement on study selection, data extraction, or quality assessment.

### Statistical analysis

The statistical analysis was accomplished by Cochrane systematic review software Review Manager (RevMan; Version 5.3, The Nordic Cochrane Centre, The Cochrane Collaboration, Copenhagen, 2014). The Mann-Whitney U-test was applied to verify the hypothesis and rendered statistical significance as z-value and *P*-value< 0·05; the results were displayed in forest plots.

The effects of the intervention on continuous outcomes, dichotomous outcomes, and time to event were expressed as mean differences or standard mean differences, odds ratios, and hazard ratios, respectively. The χ2 test with *P* < 0.1 and I2 > 50% indicated significant heterogeneity. The sensitivity analysis was performed to substitute unclear alternative decisions and ranges of values for decisions. In the presence of statistical heterogeneity, random-effects model was applied; otherwise, fixed-effects model was used.

## Results

### Study description

**Fig. 1:**
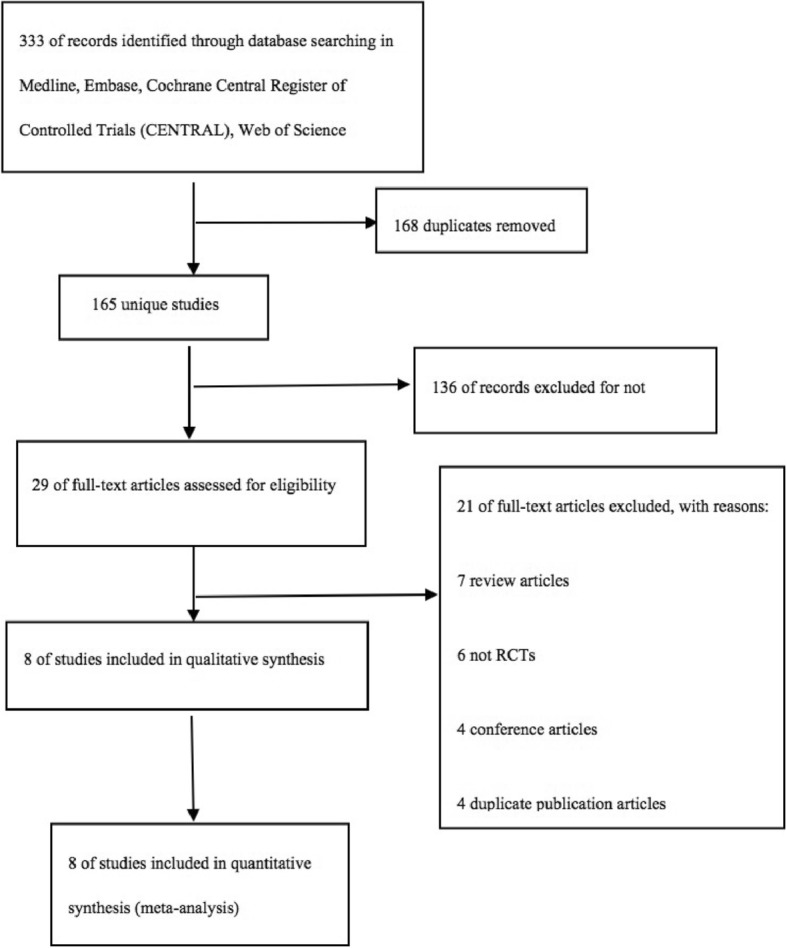
Flow diagram. CENTRAL, Cochrane Central Register of Controlled Trials; RCT, randomized controlled trial

Our search identified 333 unique studies that we assessed for eligibility, of which eight studies [11–18] with 1787 randomly assigned participants who fulfilled the eligibility criteria were enrolled in our final quantitative synthesis (Fig. 1). The eight analyzed RCTs were performed in eight different countries in three continents. Among all people, 898 patients were assigned to receive vitamin D, while 889 were administered placebo. A total of 1044 patients enrolled in the studies were male, and the male/female ratios were 535:363 and 539:360 in vitamin D and placebo groups, respectively. The mean age of patients ranged from 27.8~ 41.6 years and 26.7~ 43.7 years in the intervention and control arms, respectively. Baseline serum 25(OH)D concentrations were determined in six RCTs, ranging from 7.8~ 77.5 nmol/L and 6.0~ 79.1 nmol/L in the intervention and control arms, respectively. All studies involved the administration of vitamin D to participants in the intervention arm: this was an oral bolus dose given within a 4-month period (2.5 mg per bolus × 3 doses) in one study [18], an intramuscular bolus dose administered monthly (15 mg per bolus × 2 doses) in one study [16], an oral bolus dose administered biweekly (2.5~ 3.5 mg per bolus × 4 dose) in three studies [11–13], a combination of oral bolus does administered triweekly (1.25 mg per bolus × 3 doses) and weekly (1.25 mg per bolus × 3 doses) in one study [17], an oral bolus dose administered weekly (0.875 mg per bolus × 8 doses) in one study [14], and an oral dose administered daily (0.25 mg per day for 6 weeks) in one study [15]. Study durations ranged from 6 weeks to 8 months.

Regarding the outcome measures, six studies [11–13, 15, 16, 18] reported the proportion of sputum smear conversion, five [11–14, 17] reported the proportion of sputum culture conversion and changes of plasma calcium concentrations, four [11–14] provided the time to sputum smear conversion, four [11–13, 17] provided the time to sputum culture conversion, six [12–14, 16, 17] provided the changes in serum vitamin D concentrations, three [14, 16, 18] presented the data on TB score change, four [12, 13, 15, 16] exhibited the BMI change, four [12, 13, 16, 17] showed the changes in mean mid-upper arm circumference, five [12–16, 18] showed the changes in weight gain, three [12, 13, 16] presented the data on chest radiograph change, six [12–14, 17, 18] showed the incidence of serious adverse events, three [11, 13, 18] exhibited the incidence of non-serious adverse events, seven [11–14, 16–18] provided the incidence of all-cause deaths, three [12–14] showed the changes in blood indexes, CRP and ESR. Details on patients’ characteristics, intervention strategies, and outcomes are summarized in Tables 1 and 2.

**Table 1:** Details of each enrolled study

Author (Year)	Setting	Clinical trials register No.	Pre-protocol participants (I/C)	Participants completed study (I/C)	Intervention drug	Single dose	Frequency	Total dose	Routine	Control	Study duration	Outcomesa
Daley 2015 [11]	India	NCT00366470	247(121/126)	211(101/110)	Vitamin D3	2·5 mg	Biweekly*4 doses	10 mg	Oral	Placebo	6 weeks	①②③④⑤⑥⑮⑯
Ganmaa 2016 [12]	Mongolia	NCT01657656	390(190/200)	352(174/178)	Vitamin D3	3.5 mg	Biweekly*4 doses	13.5 mg	Oral	Placebo	8 weeks	①②③④⑤⑥⑧⑨⑩ ⑪ ⑫ ⑬ ⑭ ⑮ ⑯
Martineau 2011 [13]	UK	NCT00419068	146(73/73)	126(62/64)	Vitamin D3	2·5 mg	Biweekly*4 doses	10 mg	Oral	Placebo	8 weeks	①②③④⑤⑥⑧⑨⑩⑪⑫⑬⑭⑮⑯
Mily 2015 [14]	Bangladesh	NCT01580007	144(72/72)	128(63/65)	Vitamin D3	0.875 mg	Weekly*8 doses	7 mg	Oral	Placebo	8 weeks	②③⑤⑥⑦⑩⑫⑬⑭⑮⑯
Nursyam 2006 [15]	Indonesia	NM	67(34/33)	67(34/33)	Vitamin D3	0.25 mg	Per day*42 doses	10.5 mg	Oral	Placebo	6 weeks	①⑨⑩
Salahuddin 2013 [16]	Pakistan	NCT01130311	259(132/127)	238(119/119)	Vitamin D3	15 mg	Per month*2 doses	30 mg	Intramuscular	Placebo	8 weeks	①⑤⑦⑧⑨⑩⑪⑯
Tukvadze 2015 [17]	Georgia	NCT00918086	199(100/99)	192(97/95)	Vitamin D3	1.25 mg	Triweekly*3 doses and weekly*3 doses	7.5 mg	Oral	Placebo	16 weeks	②④⑤⑥①⑤⑦⑧⑨⑩⑪⑯
Wejse 2009 [18]	Guinea	ISRCTN35212132	355(187/178)	281(136/145)	Cholecalciferol	2.5 mg	Four-monthly*3 doses	7.5 mg	Oral	Placebo	8 months	①⑦⑩⑮⑯

^a^Outcome measures include: ①proportion of sputum smear conversion;②proportion of sputum culture conversion;③proportion of sputum smear conversion;④ proportion of sputum culture conversion;⑤ serum 25-hydroxyvitamin D concentration;⑥ serum calcium concentration;⑦ TB score;⑧ chest imaging;⑨ Body Mass Index;⑩ weight gain; ⑪ mean mid-upper arm circumference;⑫ CRP;⑬ ESR;⑭ blood cell;⑮adverse effects;⑯ mortality. *I/C* intervention/control, *NM* not mentioned, *No.* numbers, * multiply

**Table 2:** Baseline characteristics of patients in each enrolled trial

Author (Year)	Sex(male/female)	Age (Years) (mean,SD) (I/C)	Baseline chest radiograph Zones affected number(%)(I/C)	Baseline BMI (mean,SD) (I/C)	Baseline body weight (mean,SD) (I/C)	Baseline TB score (mean,SD) (I/C)	Baseline 25(OH)D (mean,SD) (nmol/L) (I/C)	Baseline calcium concentrations (mean,SD) (I/C)	Baseline CRP(mean,SD) (mg/L)(I/C)	Baseline ESR (mean,SD)(mm/h) (I/C)
Daley 2015 [11]	88/33(I) 101/25(C)	41.6 (15.1)/43.7(14.3)	NM	18.0(2.9)/17.8(3.0)	NM	NM	63.1(46.6)/62.2(51.0)	2.27(0.15)/2.28(0.17)	NM	NM
Ganmaa 2016 [12]	123/190(I) 133/200(C)	31.0 (15.6)/35.0(16.3)	7.4(4.4)/7.3(4.4)	19.7(2.8)/20.1(3.1)	NM	NM	7.8(11.8)/6.0(7.2)	2.28(0.16)/2.26(0.18)	62.7(46.1)/63.0(46.7)	17.2(10.7)/15.8(11.3)
Martineau 2011 [13]	14/48(I) 14/50(C)	30.7 (12.6)/30.5(10.1)	2.8(1.3)/2.8(1.3)	20.1(3.1)/20.2(2.7)	NM	NM	21.1(20.0)/21.3(19.0)	2.45(0.08)/2.45(0.09)	71.4(49.5)/60.5(45.0)	62.1(23.1)/60.9(17.4)
Mily 2015 [14]	36/36(I) 36/36(C)	28.1 (9.9)/26.7(8.1)	NM	NM	44.2(9.4)/43.7(7.4)	7.9(5.6)/8.0(5.0)	28.0(17.5)/28.1(16.2)	8.82(0.55)/8.65(0.59)	26.2(0.4)/32.1(0.4)	54.0(31.1)/60.2(34.9)
Nursyam 2006 [15]	20/14(I) 19/14(C)	29.9 (11.1)/32.6(11.6)	NM	16.9(2.1)/17.7(2.5)	NM	NM	NM	9.73(1.28)/9.4(0.92)	NM	31.4(5.9)/36.7(6.1)
Salahuddin 2013 [16]	71/61(I) 70/57(C)	27.8 (13.2)/28.3(14.1)	3.6(1.4)/3.6(1.5)	17.2(3.5)/17.3(4.0)	45.2(7.6)/45.6(9.0)	6.7(2.0)/6.9(2.5)	20.6(8.5)/22.9(10.3)	NM	NM	NM
Tukvadze 2015 [17]	67/33(I) 60/39(C)	34.1 (12.4)/32.4(10.6)	NM	NM	NM	NM	NM	2.11(0.3)/2.17(0.29)	NM	NM
Wejse 2009 [18]	116/71(I) 106/72(C)	37.0 (13.0)/38.0(14.0)	NM	18.8(5.3)/18.5(3.8)	51.9(9.4)/51.1(8.7)	6.7(2.1)/6.8(2.0)	77.5(23.8)/79.1(21.8)	2.03(0.26)/2.03(0.24)	NM	NM

Data reported in all patients receiving vitamin D supplementation. *BMI* body mass index, *NM* not mentioned, *No*. numbers, *SD* standard derivation, *I/C* intervention/control, *ESR* Erythrocyte sedimentation rate, *CRP* C reactive protein

Three RCTs [12–14] were at low risk of bias for all aspects analyzed. The trials conducted by Daley, Tukvadze, and Wejse as well as their colleagues had an unclear risk of attrition bias due to the missing data [11, 17, 18], which could have affected the outcome, but we found no evidence to confirm the doubt. The study conducted by Nursyam and colleagues had an unclear risk of selection, performance, and detection bias as it did not describe the definite methods used in random sequence generation, blinding of participants, and personnel and outcome assessments [15]. The study conducted by Salahuddin and colleagues had an unclear risk of performance and detection bias as it did not show the details of the blinding method [16]. Details on the risk of bias assessment are provided in Additional file 1: Figure S1 and Additional file 2: Figure S2. No studies excluding for low quality (GRADE) or dubious decisions were found in the sensitivity analysis.

### Heterogeneity

A statistical heterogeneity was not observed in the proportion of sputum smear and culture conversion, time to sputum smear and culture conversion, changes in chest radiograph, CRP, ESR, blood indexes, and number of non-serious and serious adverse events and all-cause deaths (Fig. 25; Additional file 3: Figure S3, Additional file 4: Figure S4, Additional file 5: Figure S5, Additional file 6: Figure S6, Additional file 7: Figure S7, Additional file 8: Figure S8, Additional file 9: Figure S9, Additional file 10: Figure S10, Additional file 11: Figure S11, Additional file 12: Figure S12). By contrast, a significant statistical heterogeneity was found in the changes in serum 25(OH)D, plasma calcium concentration, TB score, BMI, weight gain, mean mid-upper arm circumference, and neutrophil count (I^2^ = 99%, MD 103.36, 95%CI 84.2~ 122.53; I^2^ = 57%, MD 0.26, 95%CI 0.15~ 0.37; I^2^ = 97%, MD 0.33, 95%CI -1.58~ 2.24; I^2^ = 62%, MD 0.04, 95%CI -0.15~ 0.24; I^2^ = 60%, MD -0.21, 95%CI -0.44~ 0.01; I^2^ = 73%, MD 0.07, 95%CI -0.78~ 0.92; I^2^ = 66%, MD -0.13, 95%CI -0.42~ 0.16) (Additional file 13: Figure S13, Additional file 14: Figure S14, Additional file 15: Figure S15, Additional file 16: Figure S16, Additional file 17: Figure S17, Additional file 18: Figure S18, Additional file 19: Figure S19). To ensure that if any single study skewed the overall results, a sensitivity analysis was performed to evaluate the stability of the results. One study was removed at a time and the overall effect and summary MD recalculated. This analysis confirmed the stability of the results: the overall effects (*P* value) did not show statistically significant reversal, and summary MDs were consistent and without apparent fluctuation (range of recalculated summary MDs: 80.39~ 122.62; 0.13~ 0.33; − 0.43~ 0.72;0.02~ 0.10; − 0.23~ − 0.10; − 0.40~ 0.47;-0.18~ − 0.06).

### Outcomes

#### Primary outcomes

##### *Proportion of sputum conversion*

**Fig. 2:**
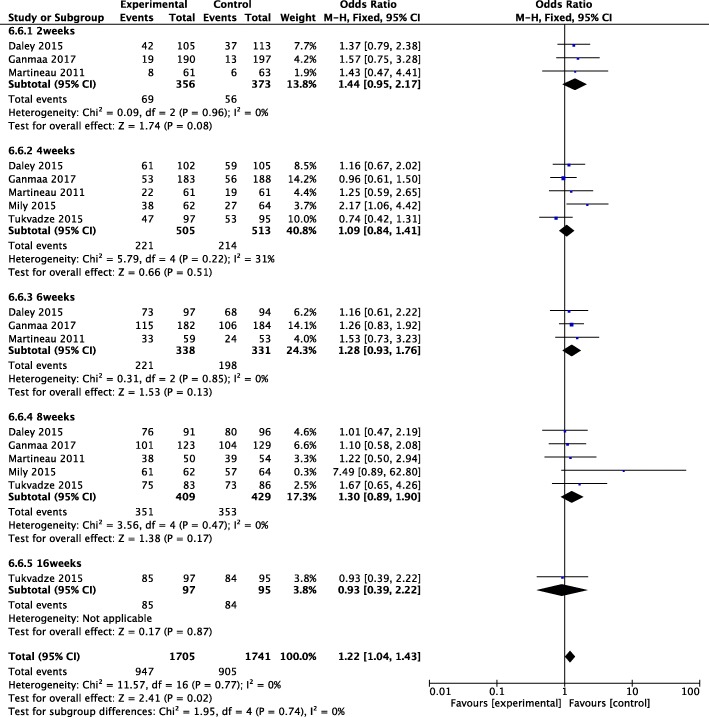
Proportion of sputum smear conversion after vitamin D supplementation. CI, confidence interval; M.-H., Mantel-Haenszel

There were significant differences in the proportion of sputum smear conversion in the overall effects (OR 1.21, 95%CI 1.05~ 1.39, *P* = 0.007), but null was found in 2, 4, 6, 8, and 12 weeks (OR 1.26, 0.93~ 1.72, *P* = 0.14; OR 1.25, 0.97~ 1.62, *P* = 0.08; OR 1.41, 1.04~ 1.92, *P* = 0.03; OR 1.02, 0.77~ 1.35, *P* = 0.90; OR 1.05, 0.56~ 1.96, *P* = 0.88) (Fig. 2).

**Fig. 3:**
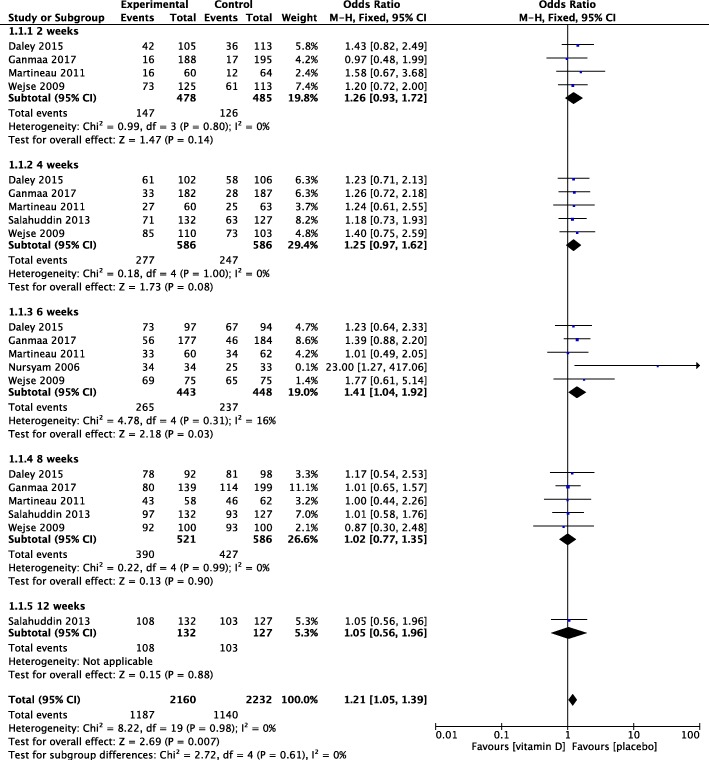
Proportion of sputum culture conversion after vitamin D supplementation. CI, confidence interval; M.-H., Mantel-Haenszel

We found significant differences in proportion of sputum culture conversion in the overall effects (OR 1.22, 1.04~ 1.43, *P* = 0.02), but null was found in 2, 4, 6, 8, and 16 weeks (OR 1.44, 0.95~ 2.17, P = 0.08; OR 1.09, 0.84~ 1.41, *P* = 0.51; OR 1.28, 0.93~ 1.76, *P* = 0.13; OR 1.30, 0.89~ 1.90, *P* = 0.17; and OR 0.93, 0.39~ 2.22, *P* = 0.87) (Fig. 3).

##### *Time to sputum conversion*

**Fig. 4:**
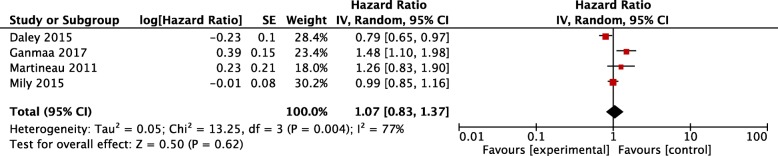
Time to sputum smear conversion after vitamin D supplementation. CI, confidence interval; SE, Standard Error; IV, Inverse Variance

**Fig. 5:**
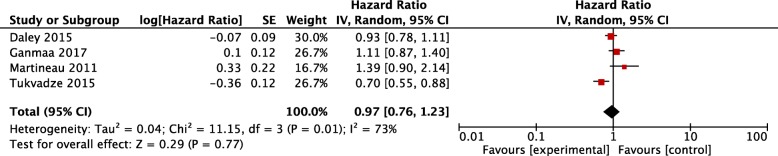
Time to sputum culture conversion after vitamin D supplementation. CI, confidence interval; SE, Standard Error; IV, Inverse Variance

Neither time to sputum smear conversion (HR 1.07, 0.83~ 1.37, *P* = 0.62) nor time to culture conversion (HR 0.97, 0.76~ 1.23, *P* = 0.77) was found with a statistical significance in vitamin D arm compared with the placebo arm (Figs. 4 and 5).

#### Secondary outcomes

##### *Hematology indexes*

We found significant differences in increase in serum 25(OH)D concentrations from baseline in 2, 4, 6, 8, 12, 16, and 24 weeks and in the overall effects (MD 103.52, 41.34~ 165.70, *P* = 0.001; MD 116.85, 60.55~ 173.16, *P* < 0.0001; MD 128.90, 124.09~ 133.71, *P* < 0.00001;MD 125.66, 76.61~ 174.72, *P* < 0.00001;MD 83.42, 26.26~ 140.58, *P* = 0.004;MD 82.51, 69.47~ 95.55, *P* < 0.00001;MD 26.50, 12.81~ 40.19, *P* = 0.0001;MD 103.36, 84.20~ 122.53, *P* < 0.00001) (Additional file 13: Figure S13).

Meanwhile, significant improvements in plasma calcium concentrations were found in the overall effects and between 4 and 6 weeks (SMD 0.26, 0.15~ 0.37, *P* < 0.00001;SMD 0.23, 0.04~ 0.43, *P* = 0.02;SMD 0.47, 0.30~ 0.65, *P* < 0.00001), but null in 2, 8, and 12 weeks (SMD 0.26, − 0.20~ 0.72, *P* = 0.27;SMD 0.24, − 0.01~ 0.48, *P* = 0.06;SMD 0.11, − 0.25~ 0.46, *P* = 0.56) (Additional file 14: Figure S14).

Significant differences were found in CRP in 2 and 6 weeks (MD 9.50, 1.94~ 17.06, *P* = 0.01;MD -7.20, − 12.79~ − 1.61,P = 0.01), but none in 4, 8, and 12 weeks and in the overall effects (MD -9.55, − 30.51~ 11.42, *P* = 0.37;MD -1.70, − 4.20~ 0.80, *P* = 0.18;MD -0.60, − 2.61~ 1.41, P = 0.56;MD -2.92, − 10.43~ 4.59, *P* = 0.45) (Additional file 4: Figure S4).

A non-significant difference was found in ESR in 2, 4, 6, 8, and 24 weeks and in the overall effects (MD 0.30, − 2.01~ 2.61, *P* = 0.80;MD 1.21, − 0.86~ 3.27, *P* = 0.25;MD -0.60, − 2.78~ 1.58, *P* = 0.59;MD 0.62, − 1.28~ 2.53, *P* = 0.52;MD -1.90, − 8.68~ 4.88, *P* = 0.58;MD 0.37, − 0.67~ 1.41, *P* = 0.48) (Additional file 5: Figure S5).

No significant difference was found in mutation in total white blood cells in 2, 4, 6, 8, and 24 weeks and in the overall effects (MD 0.40, − 0.24~ 1.04, *P* = 0.22;MD 0.20, − 0.37~ 0.77, *P* = 0.49;MD -0.20, − 0.80~ 0.40, *P* = 0.51;MD -0.29, − 0.97~ 0.40, *P* = 0.41;MD 0.32, − 0.52~ 1.16, *P* = 0.46;MD -0.02, − 0.36~ 0.32, *P* = 0.91) (Additional file 6: Figure S6).

Additionally, there was no significant difference in the changes in neutrophil count in 2, 4, 6, 8, and 24 weeks and in the overall effects (MD 0.30, − 0.31~ 0.91, *P* = 0.33;MD -0.02, − 0.33~ 0.28, *P* = 0.88;MD -0.30, − 0.87~ 0.27, *P* = 0.30;MD -0.41, − 0.92~ 0.11, *P* = 0.12;MD 0.21, − 0.19~ 0.61, *P* = 0.31;MD -0.13, − 0.42~ 0.16, *P* = 0.38) (Additional file 19: Figure S19).

No significant difference was found in alteration in monocyte count in 2, 4, 6, 8, and 24 weeks and in the overall effects (MD 0.05, − 0.01~ 0.11, *P* = 0.13;MD -0.01, − 0.06~ 0.04, *P* = 0.78;MD -0.04, − 0.01~ 0.02, *P* = 0.20;MD -0.04, − 0.12~ 0.04, *P* = 0.36;MD -0.04, − 0.15~ 0.07, *P* = 0.47;MD -0.02, − 0.05~ 0.02, *P* = 0.34) (Additional file 7: Figure S7).

A significant difference was found in the change in lymphocyte count in 4 weeks and in the overall effects (MD 0.22, 0.10~ 0.34, *P* = 0.0003;MD 0.09, 0.03~ 0.14, *P* = 0.003), while the results in 2, 6, 8, and 24 weeks (MD 0.00, − 0.12~ 0.12, *P* = 1.00;MD 0.10, − 0.03~ 0.23, P = 0.13;MD 0.05, − 0.06~ 0.15, *P* = 0.36;MD 0.02, − 0.37~ 0.41, *P* = 0.92) were on the contrary (Additional file 8: Figure S8).

##### *Imaging index*

A significant improvement in the chest radiograph (mean no. of zones involved) was detected in vitamin D arm compared with the placebo arm (MD -0.33, − 0.57~ − 0.08, *P* = 0.01) (Additional file 3: Figure S3).

##### *Clinical indexes*

No significant improvement was found in TB score in 8 weeks and 5, 6, and 8 months (MD -0.01, − 0.23~ 0.22, *P* = 0.98; MD -0.09, − 0.26~ 0.08, *P* = 0.29; MD 0.01, − 1.69~ 1.71, *P* = 0.99; MD -0.18, − 0.36~ 0.00, *P* = 0.05; MD -0.21, − 0.44~ 0.01, *P* = 0.07), except 12 weeks (MD -1.18, − 1.78~ 0.59, *P* < 0.0001) (Additional file 15: Figure S15). No significant difference was found in the change in hemoglobin levels in 4, 8, and 24 weeks and in the overall effects (MD -0.13, − 0.77~ 0.51, *P* = 0.69; MD -0.10, − 0.51~ 0.31, *P* = 0.64; MD -0.25, − 0.96~ 0.46, *P* = 0.49; MD -0.13, − 0.45~ 0.18, *P* = 0.40) (Additional file 9: Figure S9). An insignificant improvement was found in the anthropometric outcomes: BMI, weight gain and mean mid-upper arm circumference (MD 0.33, − 1.58~ 2.24, *P* = 0.74; SMD 0.04, − 0.15~ 0.24, *P* = 0.68; MD 0.07, − 0.78~ 0.92, *P* = 0.88) (Additional file 16: Figure S16, Additional file 17: Figure S17, Additional file 18: Figure S18).

##### Safety and mortality

Pooled analysis showed that no significant difference was found in the number of non-serious adverse events, serious adverse events, and all-cause deaths (OR 1.06, 0.65~ 1.74, *P* = 0.80; OR 1.02, 0.48~ 2.20, *P* = 0.95; OR 1.22, 0.74~ 2.04, *P* = 0.43) (Additional file 10: Figure S10, Additional file 11: Figure S11, Additional file 12: Figure S12).

## Discussion

This is the largest and the most comprehensive meta-analysis to investigate the effects of adjunctive vitamin D on patients with PTB currently. In this meta-analysis, we found that vitamin D could increase the proportion of sputum smear and culture conversion, but was unable to shorten the time to sputum smear and culture conversion. An increase in lymphocyte count, serum 25(OH)D, and plasma calcium concentrations and improvement in chest radiograph were observed after vitamin D supplementation. There was no evidence of improvement of other parameters (TB score, BMI, mean mid-upper arm circumference, weight gain, CRP, ESR, and blood indexes) in the vitamin D arm. No significant difference was found in the safety (non-serious and serious adverse events) and mortality (all-cause deaths) between two groups.

The results of this meta-analysis are inconsistent with those of previous meta-analyses, showing that vitamin D supplementation safely and effectively increases the proportion of sputum smear and culture conversion. A meta-analysis that included four trials did not show any improvement in the clinical parameters of the participants in the vitamin D arm compared with those in the placebo arm (*P* = 0.05) [7]. Unfortunately, no further information was obtained from this congress literature after contacting the corresponding author. Another meta-analysis, which included five studies (one study aimed at children) with 841 participants, showed that vitamin D supplementation does not have any beneficial effects on improving sputum smear and culture conversion, adverse effects, and body weight [8]. These two meta-analyses had limited number of studies, sample sizes, and parameters analyzed, influencing the results.

Vitamin D supplementation as adjunctive therapy had no impact on the time to sputum smear or culture conversion, the latter is recognized as a surrogate endpoint for treatment failure and relapse in patients with TB [19], despite the increase in the proportion of sputum smear and culture conversion. Thus, more rigorous and larger scale RCTs should be conducted to further verify this problem.

Besides, we detected whether adjunctive vitamin D exhibited anti-inflammatory actions on influencing the peripheral blood parameters. A significant increase in lymphocyte count was observed in the vitamin D arm, but null was found in total white blood cell count, neutrophil count, monocyte count, CRP, and ESR. The lymphocyte: monocyte ratio was verified as a biomarker of pulmonary inflammation resolution in an animal model of TB [20]. Coussens et al. [21] found that adjunctive vitamin D therapy accelerated sputum smear conversion as well as enhanced resolution of lymphopaenia and monocytosis in PTB patients. In our meta-analysis, improvements of sputum conversion and lymphocyte: monocyte ratio were compliance with previous studies. These findings propose a potential effect of vitamin D supplementation on accelerating resolution of inflammatory responses during tuberculosis therapy. Thus, we speculated that the modest immune modulatory effect of vitamin D might have a preventing role on TB [22]; this hypothesis is being solved by two ongoing clinical trials [23, 24].

Statistical differences in the improvement of chest imaging were observed between groups, but studies included in the analysis are less. However, this result needs to be verified further. A non-statistical difference in BMI, weight gain, mean mid-upper arm circumference, hemoglobin, and TB scores was observed among patients receiving vitamin D supplements compared with those in the placebo group. The indicator for weight gain used in this study was consistent with that in the previous meta-analyses.

Based on the presently available studies, serum 25(OH)D and plasma calcium concentrations returned to normal after vitamin D administration. The mostly reported non-serious adverse event is hypocalcemia. Adverse events and all-cause deaths were evenly distributed between the vitamin D and placebo groups. Results confirmed that vitamin D supplementation is safe and effective. These findings are accord with previous meta-analyses.

This study has several strengths. Our meta-analysis has the largest number of studies and participants currently, and the studies included were of high quality. This study is the first meta-analysis to examine the effects of vitamin D supplementation on the time to sputum smear or culture conversion; the latter was considered a surrogate endpoint for treatment failure and relapse [19]. Besides, 25(OH)D concentration was measured using validated assays in laboratories. The proportion of missing outcome data was small and similar in both groups. Therefore, our findings have a high degree of validity.

This study has some limitations. The administration doses of vitamin D, duration of treatment, and follow-up time were different. Heterogeneity was found in some analyses (serum 25(OH)D, plasma calcium concentration, BMI, weight gain, TB score, mean mid-upper arm circumference, and neutrophil count). For small number of published RCTs, power of some analyses was limited. These may affect the accuracy of analysis results.

Genetic variation in the gene encoding the vitamin D receptor (vitamin D receptor polymorphisms) may modify the effects of adjunctive vitamin D in PTB. Jolliffe et al. [25] suggested that single nucleotide polymorphisms(SNPs) in special gene in the vitamin D pathway have an influence on disease outcomes. Ganmaa et al. [12] showed that vitamin D_3_ did not improve time to sputum culture conversion overall, but significant difference was found in patients with one or more minor alleles for SNPs. So, we speculate that vitamin D receptor polymorphisms may affect the effects of vitamin D adjunctive treatment. But unfortunately, we cannot do subgroup analysis in our meta-analysis with insufficient data of relevant studies.

For low cost of this intervention and major economic burden of PTB, vitamin D is regarded as a cost-effective adjunctive therapy.

## Conclusions

In conclusion, vitamin D supplementation can safely and effectively increase the proportion of sputum smear and culture conversion, but it may not have enough beneficial effects on time to sputum conversion. As this intervention is safe and low cost, it is considered a cost-effective strategy in treating patients with PTB. Thus, future rigorous RCTs are needed, particularly with different vitamin D dose, treatment duration, and follow-up based on vitamin D receptor polymorphisms and severities of diseases, to further determine the role of vitamin D in patients with PTB.

## Additional files

Additional file 1: **Figure S1.** Risk of bias graph. (PDF 80 kb)

Additional file 2: **Figure S2.** Risk of bias summary. (PDF 96 kb)

Additional file 3: **Figure S3.** Change of chest radiograph after vitamin D supplementation. CI, confidence interval; SD, standard derivation; IV, Inverse Variance. (PDF 101 kb)

Additional file 4: **Figure S4.** Change of CRP after vitamin D supplementation. CI, confidence interval; SD, standard derivation; IV, Inverse Variance. (PDF 311 kb)

Additional file 5: **Figure S5.** Change of ESR after vitamin D supplementation. CI, confidence interval; SD, standard derivation; IV, Inverse Variance. (PDF 102 kb)

Additional file 6: **Figure S6.** Change of white blood cell count after vitamin D supplementation. CI, confidence interval; SD, standard derivation; IV, Inverse Variance. (PDF 302 kb)

Additional file 7: **Figure S7.** Change of monocyte count after vitamin D supplementation. CI, confidence interval; SD, standard derivation; IV, Inverse Variance. (PDF 301 kb)

Additional file 8: **Figure S8.** Change of lymphocyte count after vitamin D supplementation. CI, confidence interval; SD, standard derivation; IV, Inverse Variance. (PDF 293 kb)

Additional file 9: **Figure S9.** Change of hemoglobin count after vitamin D supplementation. CI, confidence interval; SD, standard derivation; IV, Inverse Variance. (PDF 195 kb)

Additional file 10: **Figure S10.** Non-serious adverse events after vitamin D supplementation. CI, confidence interval; M.-H., Mantel-Haenszel. (PDF 91 kb)

Additional file 11: **Figure S11.** Serious adverse events after vitamin D supplementation. CI, confidence interval; M.-H., Mantel-Haenszel. (PDF 110 kb)

Additional file 12: **Figure S12.** All-cause deaths after vitamin D supplementation. CI, confidence interval; M.-H., Mantel-Haenszel. (PDF 124 kb)

Additional file 13: **Figure S13**. Serum 25(OH)D concentration after vitamin D supplementation. CI, confidence interval; SD, standard derivation; IV, Inverse Variance. (PDF 502 kb)

Additional file 14: **Figure S14**. Plasma calcium concentration after vitamin D supplementation. CI, confidence interval; SD, standard derivation; IV, Inverse Variance. (PDF 399 kb)

Additional file 15: **Figure S15**. TB score after vitamin D supplementation. CI, confidence interval; SD, standard derivation; IV, Inverse Variance. (PDF 291 kb)

Additional file 16: **Figure S16**. Change of Body-Mass Index after vitamin D supplementation. CI, confidence interval; SD, standard derivation; IV, Inverse Variance. (PDF 116 kb)

Additional file 17: **Figure S17**. Weight gain after vitamin D supplementation. CI, confidence interval; SD, standard derivation; IV, Inverse Variance. (PDF 141 kb)

Additional file 18: **Figure S18**. Change of Mean Mid-Upper Arm Circumference after vitamin D supplementation. CI, confidence interval; SD, standard derivation; IV, Inverse Variance. (PDF 92 kb)

Additional file 19: **Figure S19.** Change of neutrophil count after vitamin D supplementation. CI, confidence interval; SD, standard derivation; IV, Inverse Variance. (PDF 297 kb)

## Abbreviations

25(OH)D: 25-hydroxy vitamin D
BMI: Body mass index
CRP: C-reactive protein
ESR: Erythrocyte sedimentation rate
PTB: Pulmonary tuberculosis
SNPs: single nucleotide polymorphisms
TB: Tuberculosis
