# Supplementary figures and images for: Effects of vitamin D supplementation on the outcomes of patients with pulmonary tuberculosis: a systematic review and meta-analysis
Source: BMC Pulm Med. 2018 Jun 28;18:108. doi: 10.1186/s12890-018-0677-6 (PMC6025740; doi:10.1186/s12890-018-0677-6)

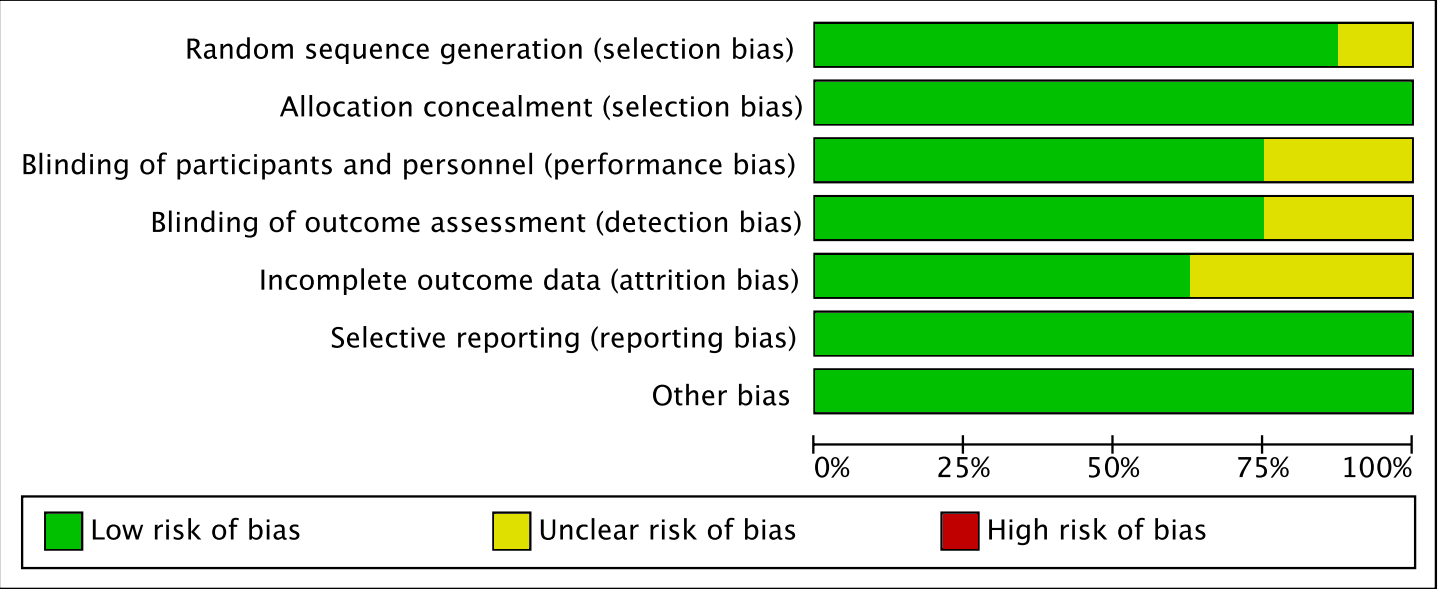

Supplement: Supplementary file 1 — Figure S1. Risk of bias graph. (PDF 80 kb) [file 12890_2018_677_MOESM1_ESM.pdf]

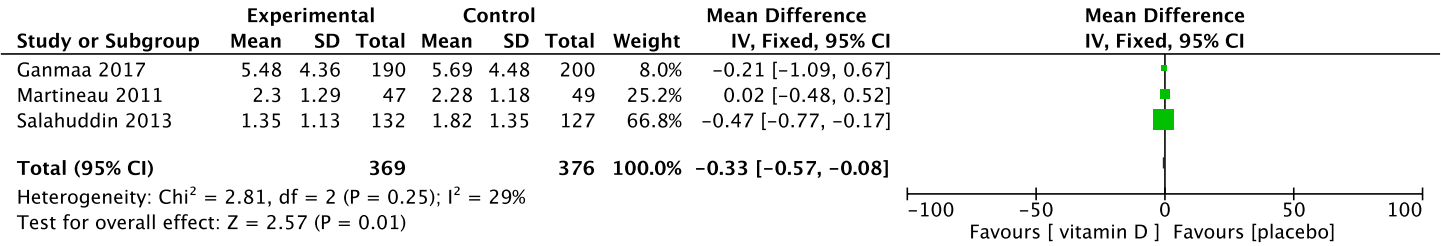

Supplement: Supplementary file 3 — Figure S3. Change of chest radiograph after vitamin D supplementation. CI, confidence interval; SD, standard derivation; IV, Inverse Variance. (PDF 101 kb) [file 12890_2018_677_MOESM3_ESM.pdf]

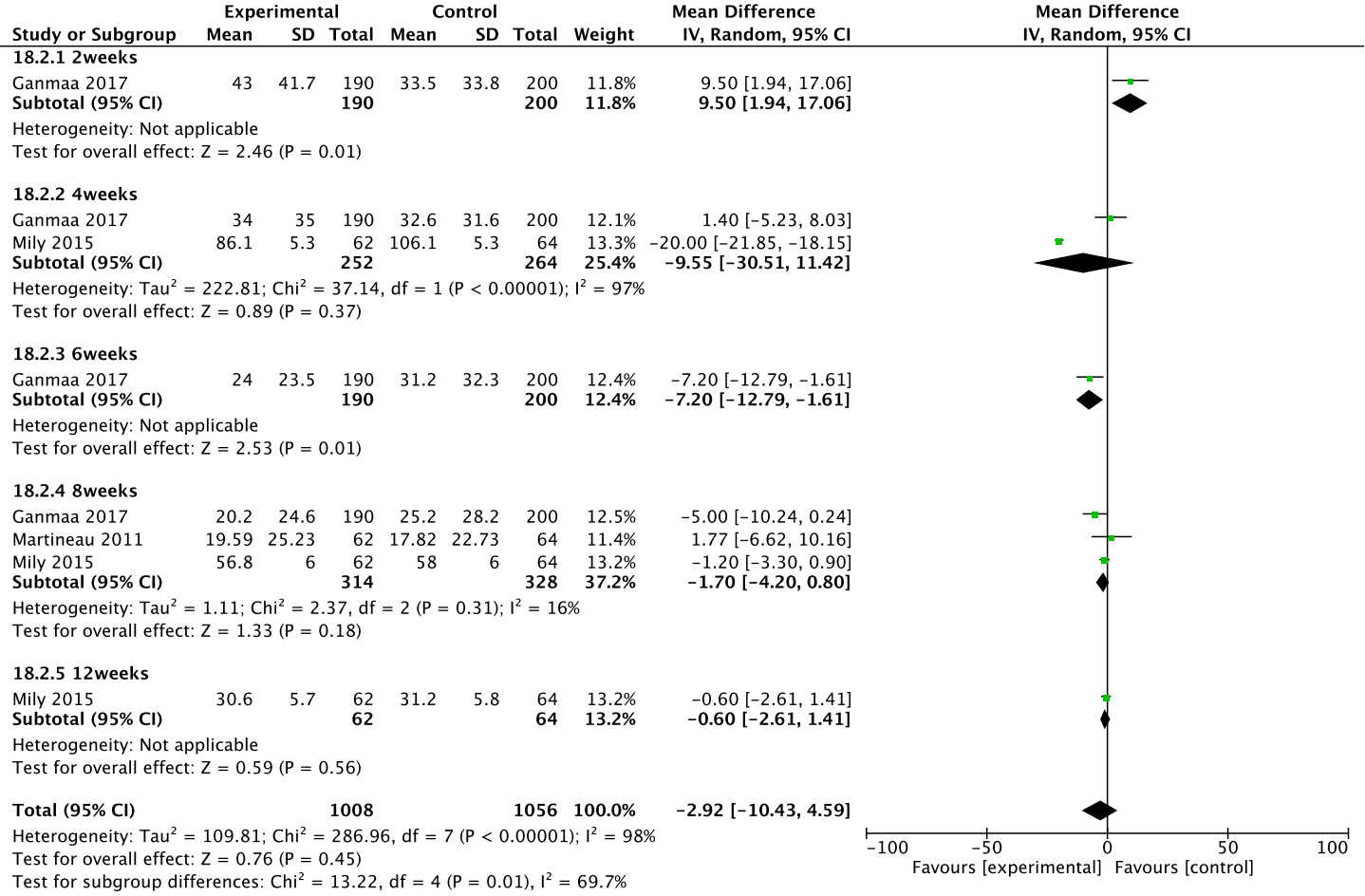

Supplement: Supplementary file 4 — Figure S4. Change of CRP after vitamin D supplementation. CI, confidence interval; SD, standard derivation; IV, Inverse Variance. (PDF 311 kb) [file 12890_2018_677_MOESM4_ESM.pdf]

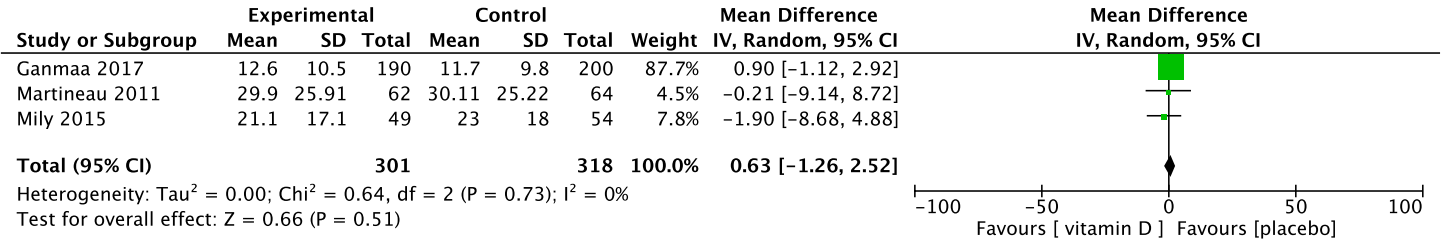

Supplement: Supplementary file 5 — Figure S5. Change of ESR after vitamin D supplementation. CI, confidence interval; SD, standard derivation; IV, Inverse Variance. (PDF 102 kb) [file 12890_2018_677_MOESM5_ESM.pdf]

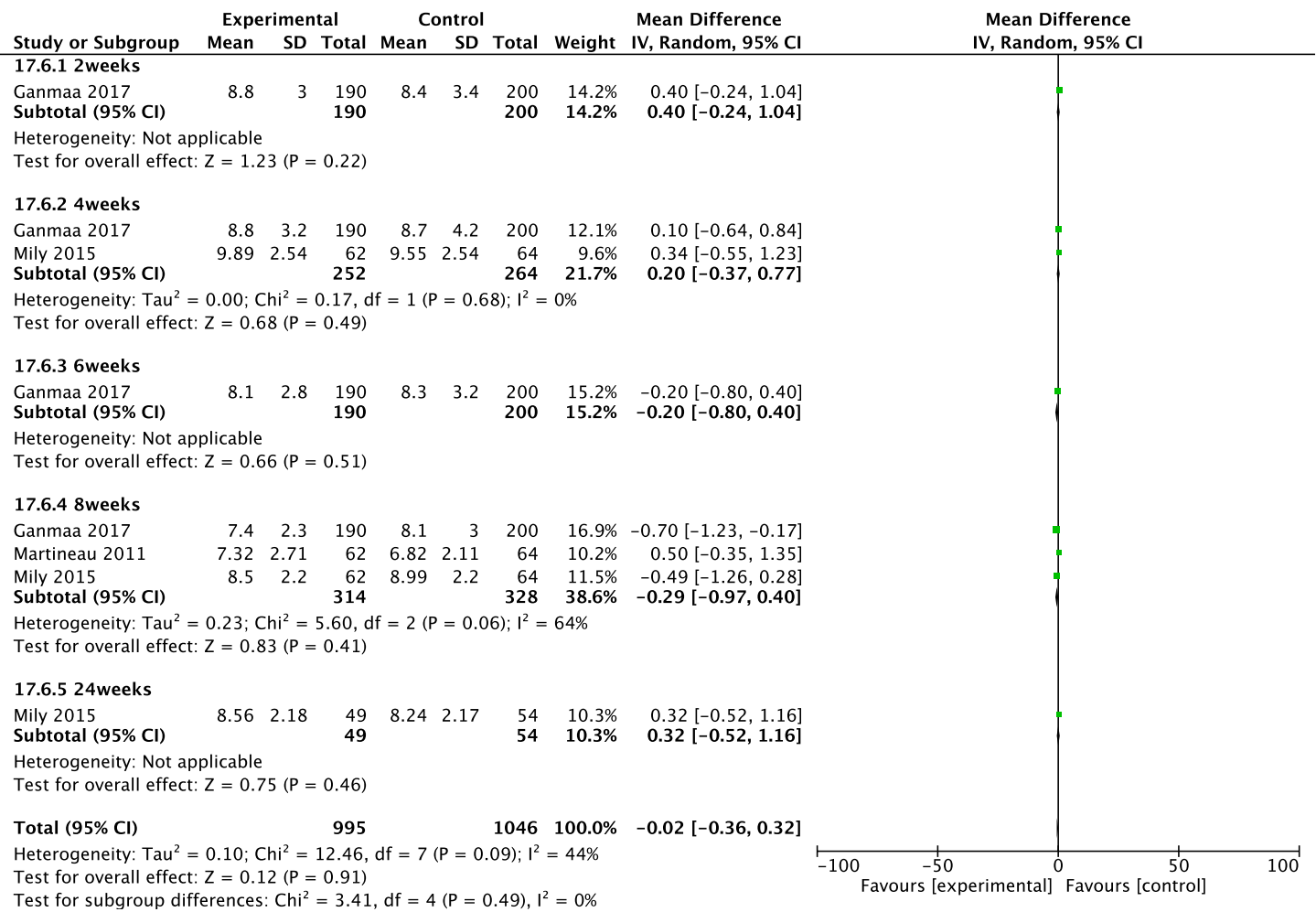

Supplement: Supplementary file 6 — Figure S6. Change of white blood cell count after vitamin D supplementation. CI, confidence interval; SD, standard derivation; IV, Inverse Variance. (PDF 302 kb) [file 12890_2018_677_MOESM6_ESM.pdf]

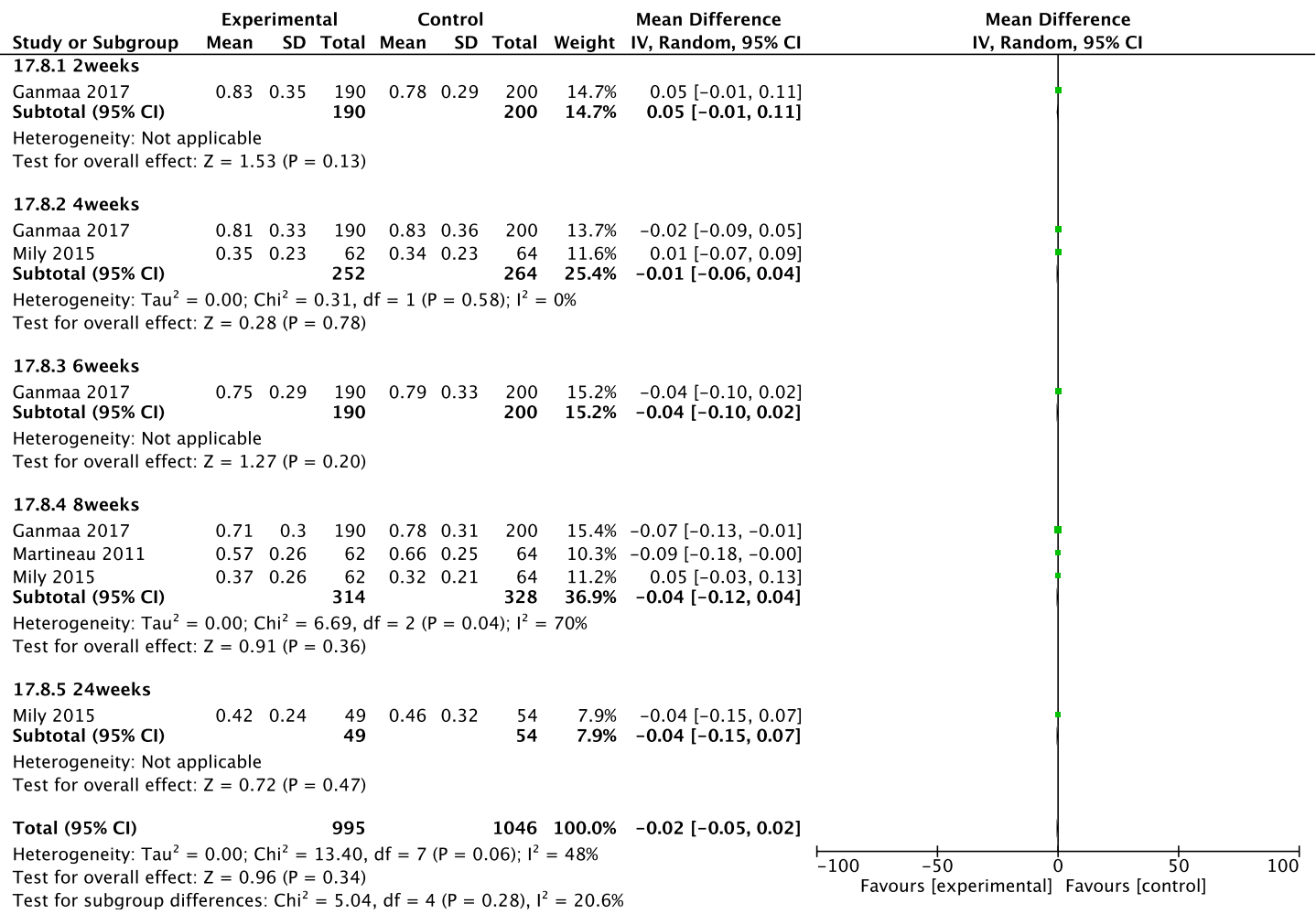

Supplement: Supplementary file 7 — Figure S7. Change of monocyte count after vitamin D supplementation. CI, confidence interval; SD, standard derivation; IV, Inverse Variance. (PDF 301 kb) [file 12890_2018_677_MOESM7_ESM.pdf]

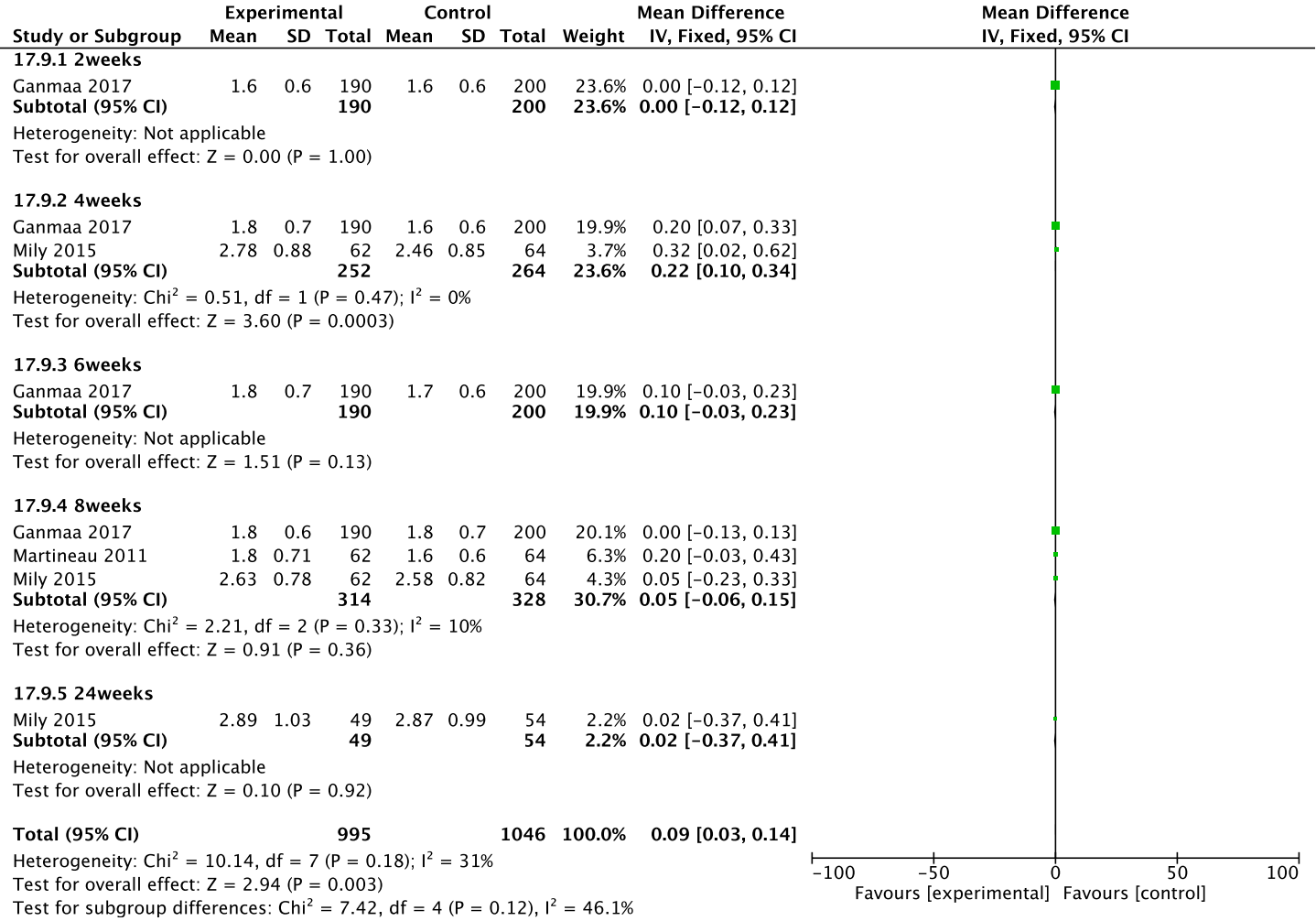

Supplement: Supplementary file 8 — Figure S8. Change of lymphocyte count after vitamin D supplementation. CI, confidence interval; SD, standard derivation; IV, Inverse Variance. (PDF 293 kb) [file 12890_2018_677_MOESM8_ESM.pdf]

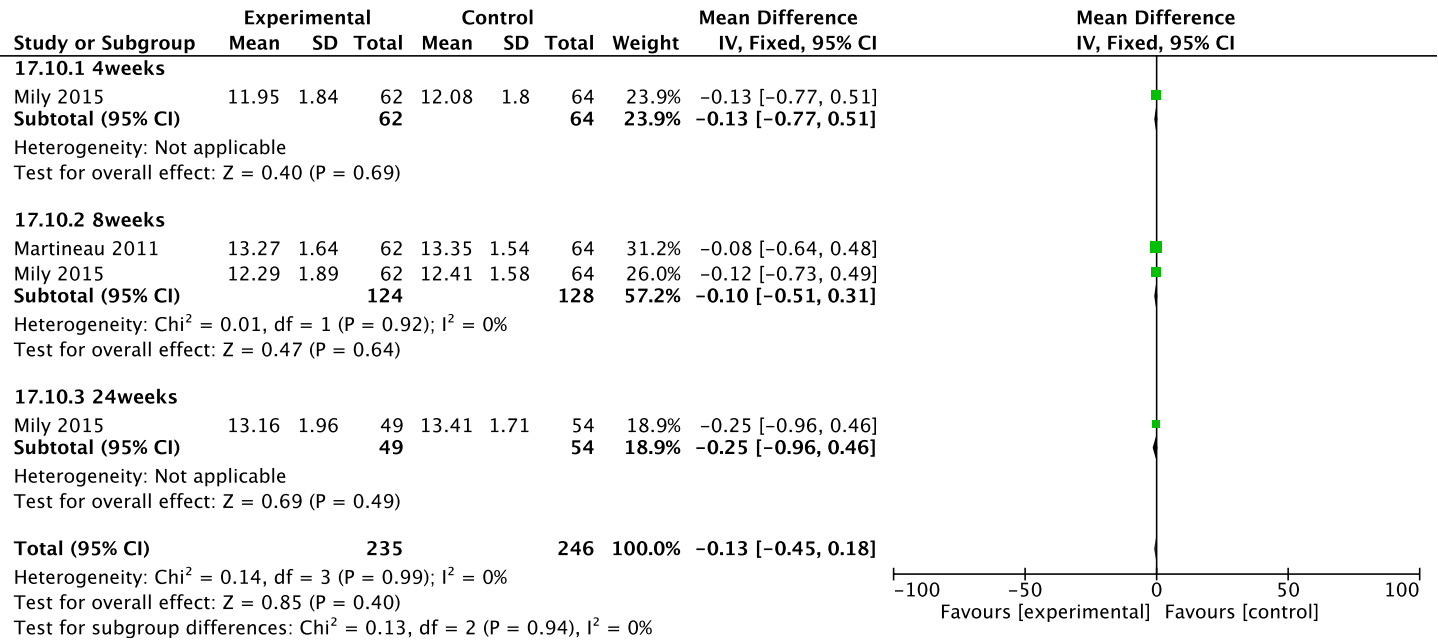

Supplement: Supplementary file 9 — Figure S9. Change of hemoglobin count after vitamin D supplementation. CI, confidence interval; SD, standard derivation; IV, Inverse Variance. (PDF 195 kb) [file 12890_2018_677_MOESM9_ESM.pdf]

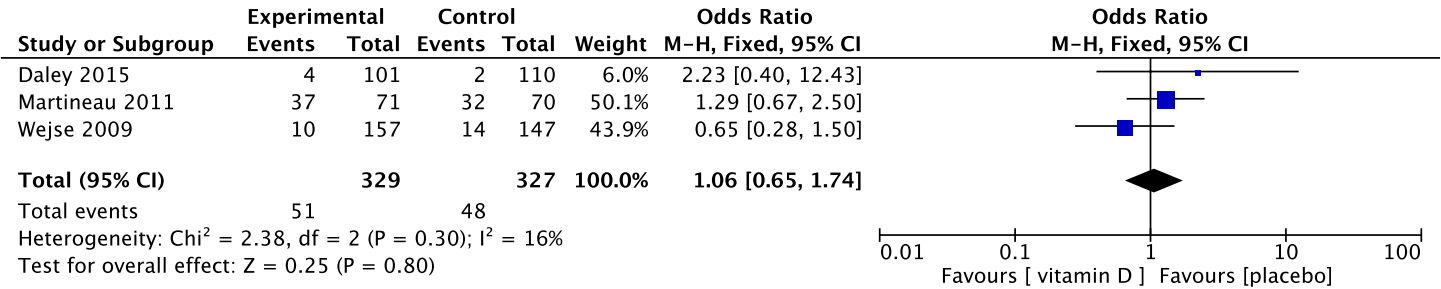

Supplement: Supplementary file 10 — Figure S10. Non-serious adverse events after vitamin D supplementation. CI, confidence interval; M.-H., Mantel-Haenszel. (PDF 91 kb) [file 12890_2018_677_MOESM10_ESM.pdf]

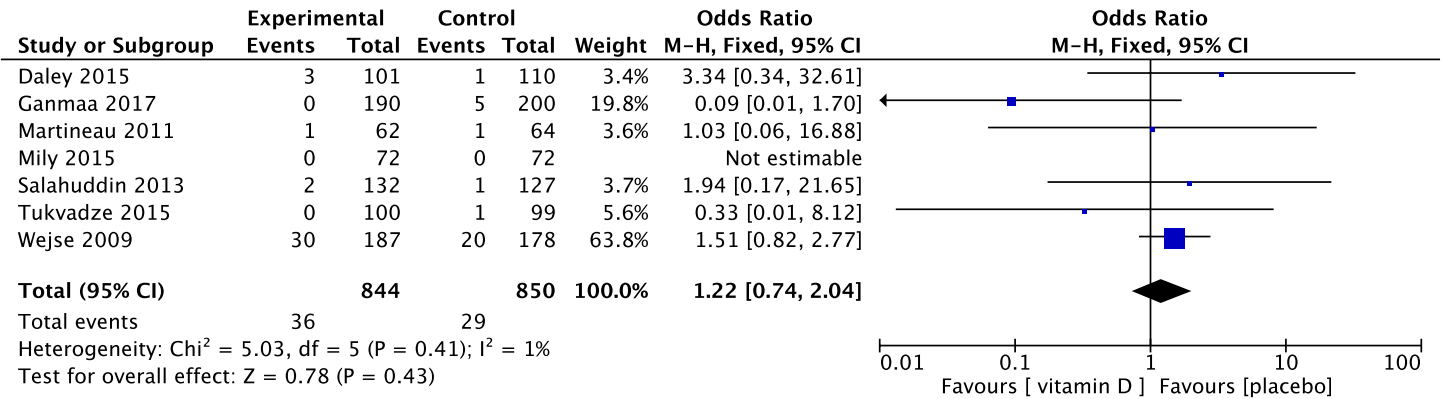

Supplement: Supplementary file 12 — Figure S12. All-cause deaths after vitamin D supplementation. CI, confidence interval; M.-H., Mantel-Haenszel. (PDF 124 kb) [file 12890_2018_677_MOESM12_ESM.pdf]

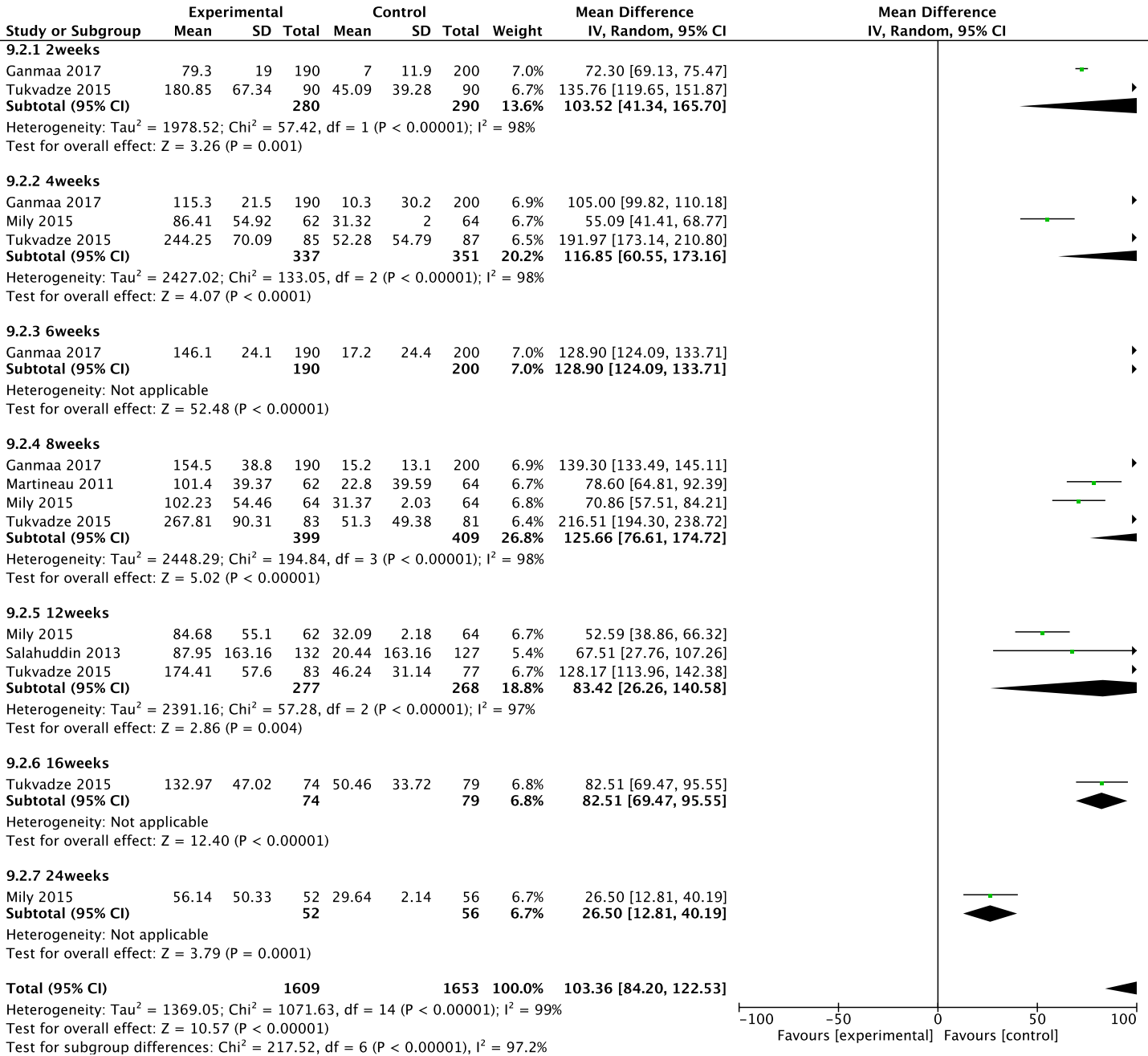

Supplement: Supplementary file 13 — Figure S13. Serum 25(OH)D concentration after vitamin D supplementation. CI, confidence interval; SD, standard derivation; IV, Inverse Variance. (PDF 502 kb) [file 12890_2018_677_MOESM13_ESM.pdf]

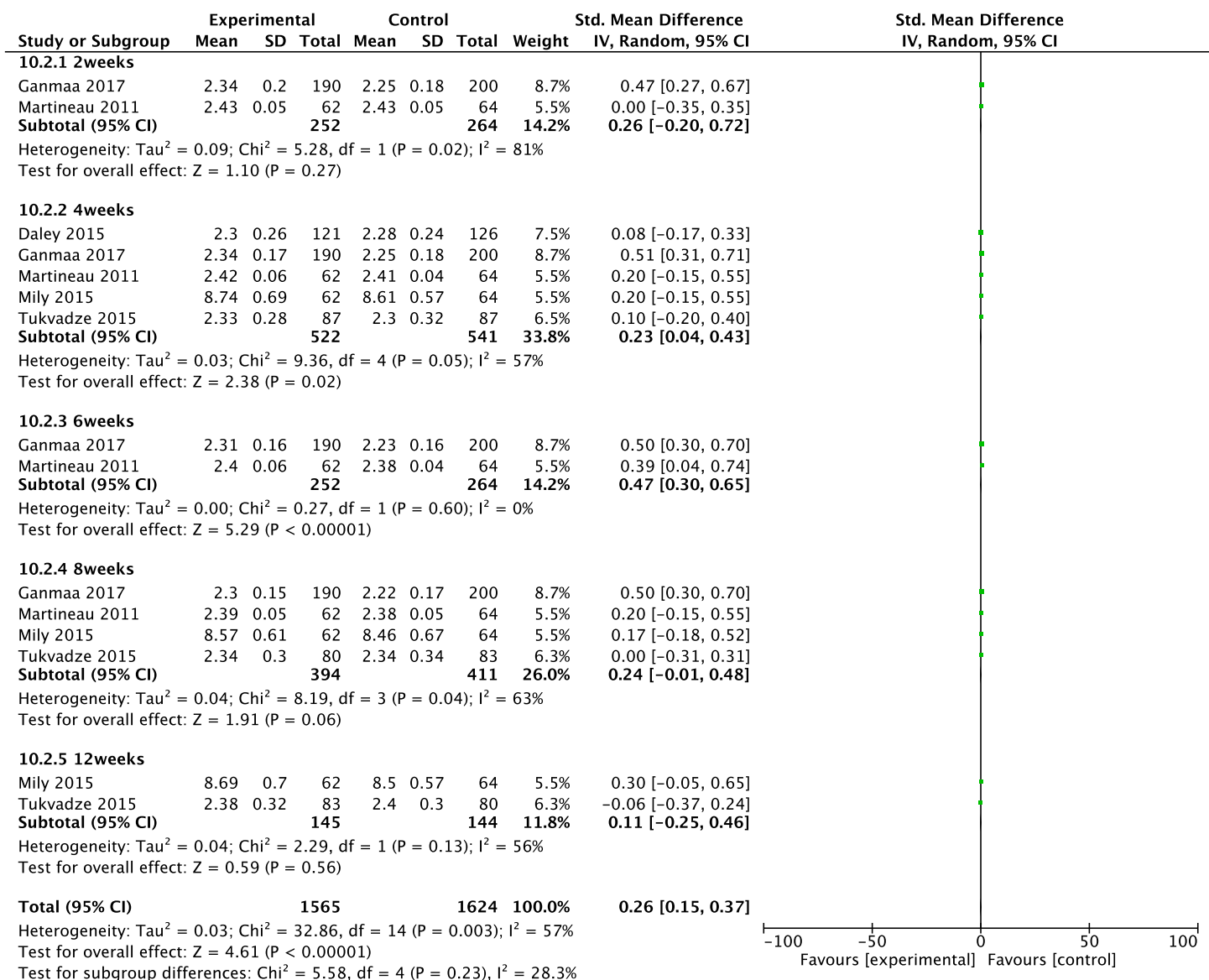

Supplement: Supplementary file 14 — Figure S14. Plasma calcium concentration after vitamin D supplementation. CI, confidence interval; SD, standard derivation; IV, Inverse Variance. (PDF 399 kb) [file 12890_2018_677_MOESM14_ESM.pdf]

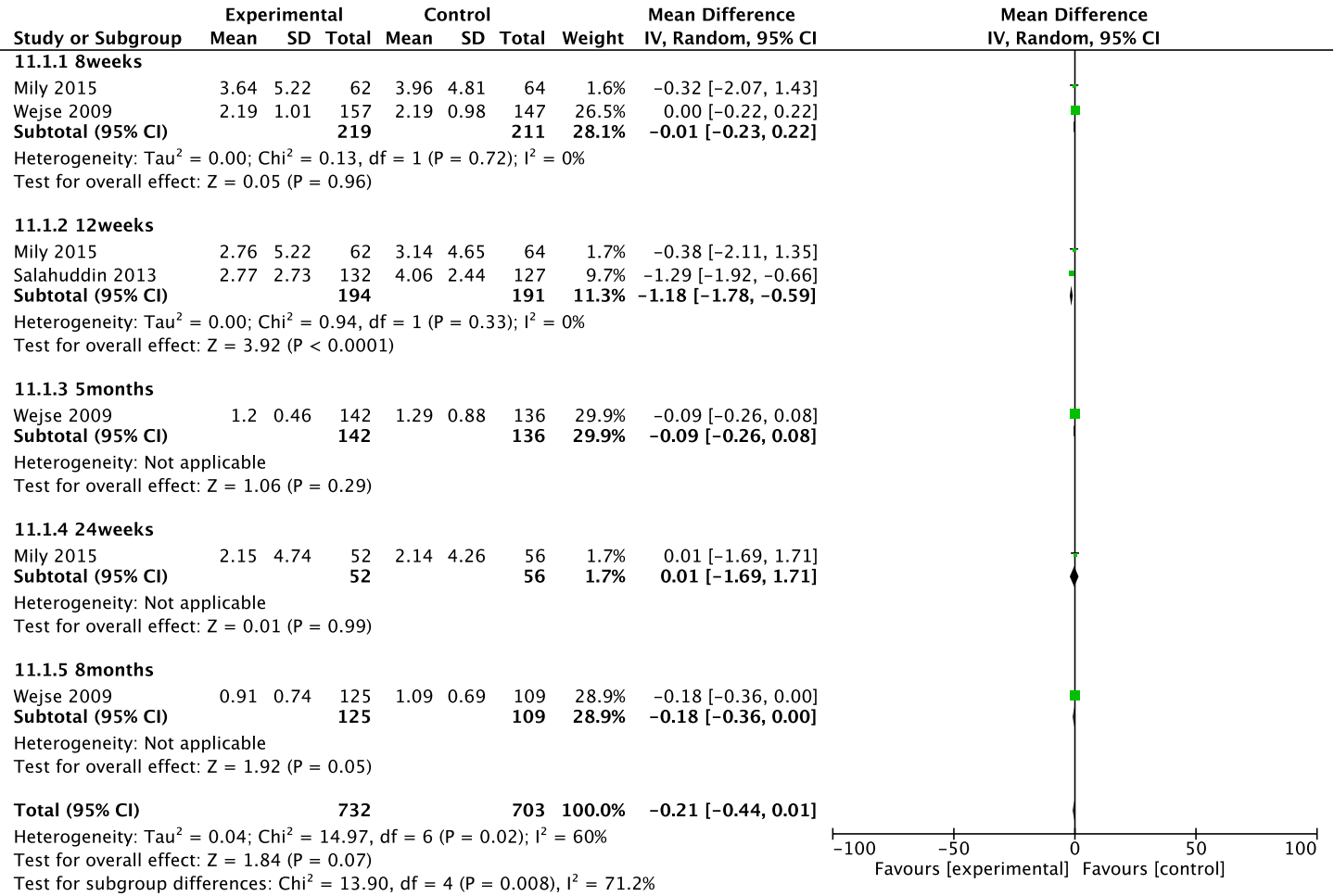

Supplement: Supplementary file 15 — Figure S15. TB score after vitamin D supplementation. CI, confidence interval; SD, standard derivation; IV, Inverse Variance. (PDF 291 kb) [file 12890_2018_677_MOESM15_ESM.pdf]

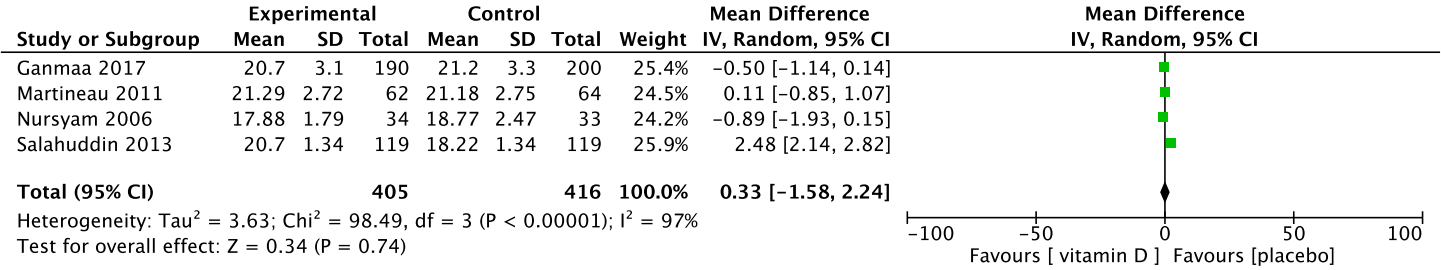

Supplement: Supplementary file 16 — Figure S16. Change of Body-Mass Index after vitamin D supplementation. CI, confidence interval; SD, standard derivation; IV, Inverse Variance. (PDF 116 kb) [file 12890_2018_677_MOESM16_ESM.pdf]

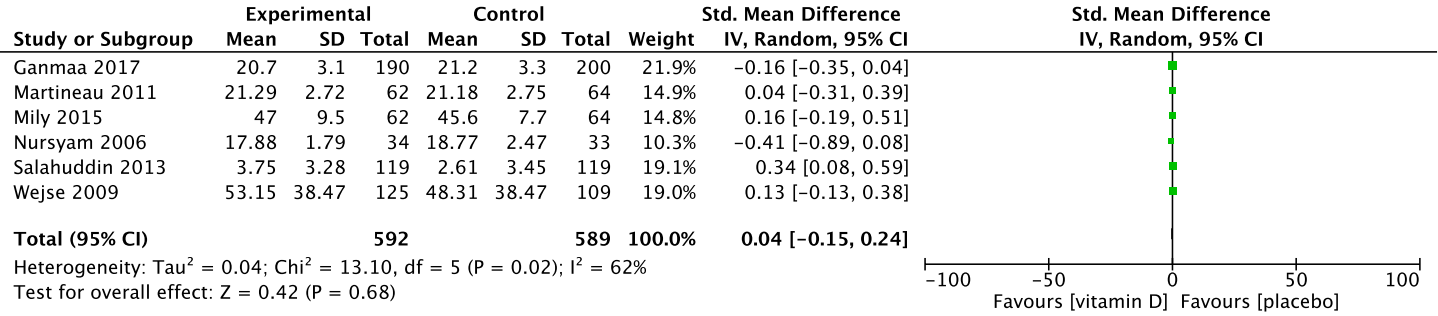

Supplement: Supplementary file 17 — Figure S17. Weight gain after vitamin D supplementation. CI, confidence interval; SD, standard derivation; IV, Inverse Variance. (PDF 141 kb) [file 12890_2018_677_MOESM17_ESM.pdf]

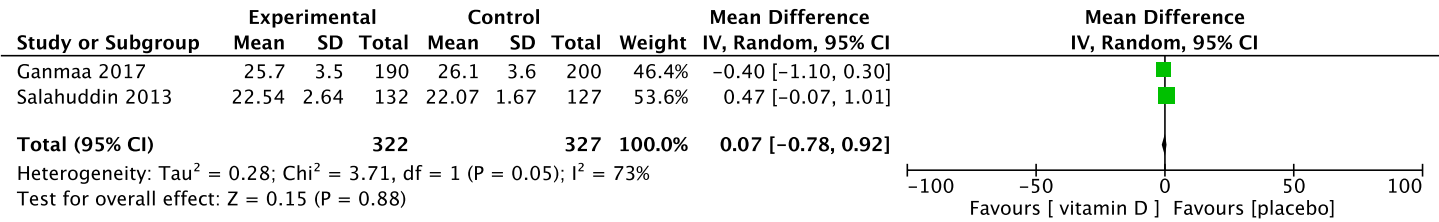

Supplement: Supplementary file 18 — Figure S18. Change of Mean Mid-Upper Arm Circumference after vitamin D supplementation. CI, confidence interval; SD, standard derivation; IV, Inverse Variance. (PDF 92 kb) [file 12890_2018_677_MOESM18_ESM.pdf]

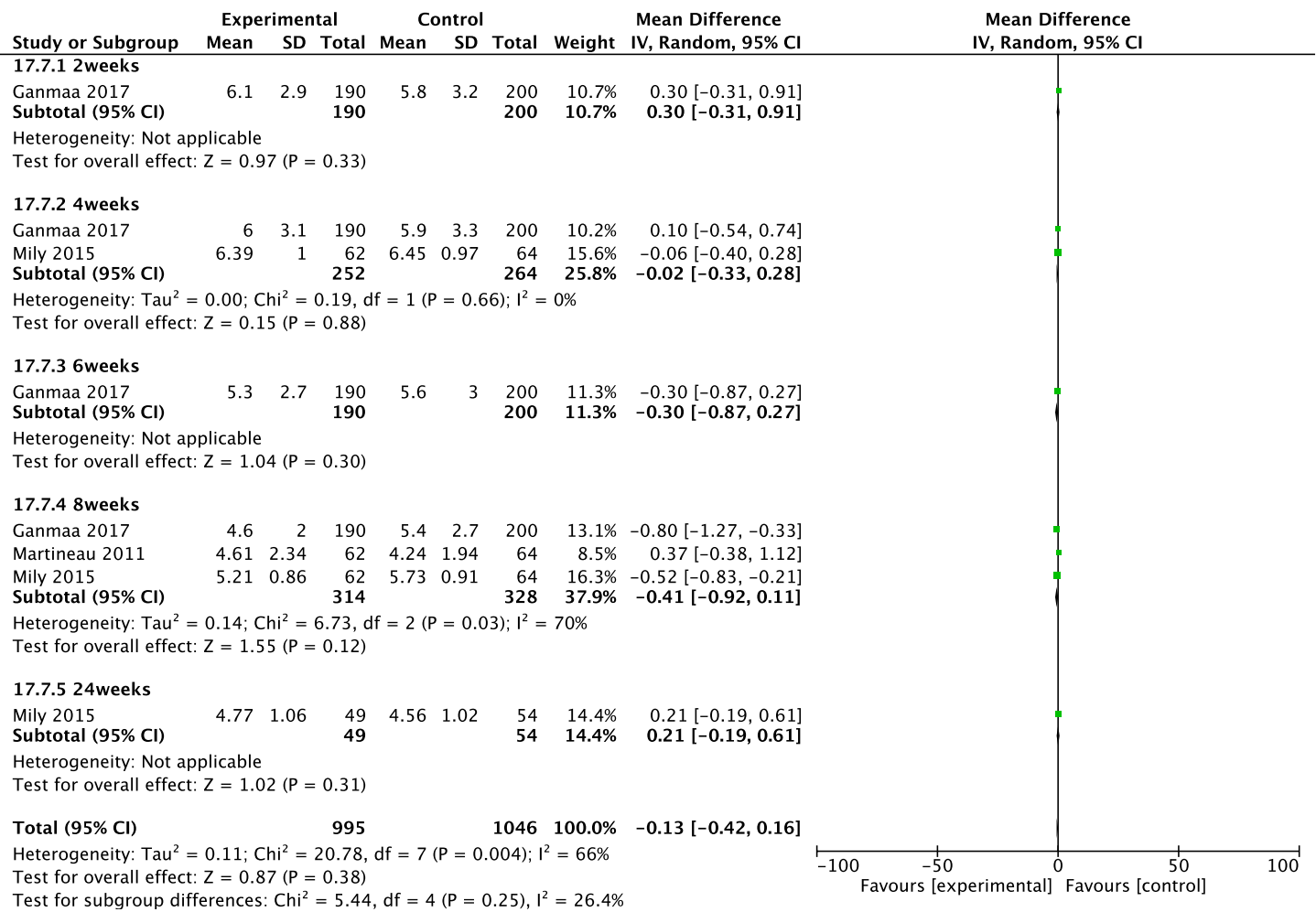

Supplement: Supplementary file 19 — Figure S19. Change of neutrophil count after vitamin D supplementation. CI, confidence interval; SD, standard derivation; IV, Inverse Variance. (PDF 297 kb) [file 12890_2018_677_MOESM19_ESM.pdf]
