# Supplementary material for: Effects of vitamin D supplementation on the outcomes of patients with pulmonary tuberculosis: a systematic review and meta-analysis
Source: BMC Pulm Med. 2018 Jun 28;18:108. doi: 10.1186/s12890-018-0677-6 (PMC6025740; doi:10.1186/s12890-018-0677-6)
Supplement: Supplementary file 2 — Figure S2. Risk of bias summary. (PDF 96 kb) [file 12890_2018_677_MOESM2_ESM.pdf]

	Random sequence generation (selection bias)	Allocation concealment (selection bias)	Blinding of participants and personnel (performance bias)	Blinding of outcome assessment (detection bias)	Incomplete outcome data (attrition bias)	Selective reporting (reporting bias)	Other bias
Daley 2015	+	+	+	+	?	+	+
Ganmaa 2017	+	+	+	+	+	+	+
Martineau 2011	+	+	+	+	+	+	+
Mily 2015	+	+	+	+	+	+	+
Nursyam 2006	?	+	?	?	+	+	+
Salahuddin 2013	+	+	?	?	+	+	+
Tukvadze 2015	+	+	+	+	?	+	+
Wejse 2009	+	+	+	+	?	+	+
