# Supplementary material for: Effects of vitamin D supplementation on the outcomes of patients with pulmonary tuberculosis: a systematic review and meta-analysis
Source: BMC Pulm Med. 2018 Jun 28;18:108. doi: 10.1186/s12890-018-0677-6 (PMC6025740; doi:10.1186/s12890-018-0677-6)

Study or Subgroup	Experimental Events	Experimental Total	Control Events	Control Total	Weight	Odds Ratio M-H, Fixed, 95% CI
Daley 2015	3	101	2	110	14.4%	1.65 [0.27, 10.10]
Ganmaa 2017	1	190	5	200	37.5%	0.21 [0.02, 1.78]
Martineau 2011	7	71	2	70	14.0%	3.72 [0.74, 18.57]
Mily 2015	0	62	1	64	11.3%	0.34 [0.01, 8.47]
Tukvadze 2015	2	100	3	99	22.8%	0.65 [0.11, 4.00]
Wejse 2009	0	187	0	178		Not estimable
**Total (95% CI)**		**711**		**721**	**100.0%**	**1.02 [0.48, 2.20]**
Total events	13		13			

Heterogeneity: $Chi^2 = 5.55$, df = 4 (P = 0.24); $I^2 = 28\%$
Test for overall effect: Z = 0.06 (P = 0.95)

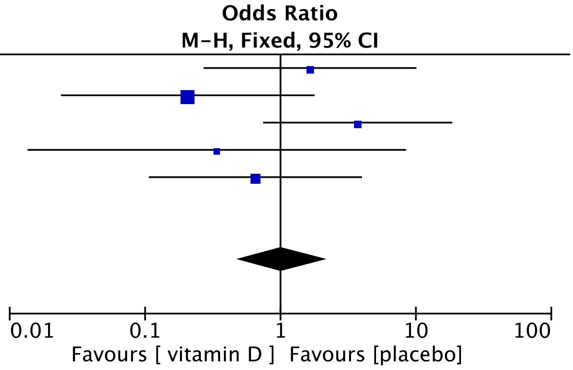

Supplement: Supplementary file 11 — Figure S11. Serious adverse events after vitamin D supplementation. CI, confidence interval; M.-H., Mantel-Haenszel. (PDF 110 kb) [file 12890_2018_677_MOESM11_ESM.pdf]
